# Vitamin D Status and Mortality from SARS CoV-2: A Prospective Study of Unvaccinated Caucasian Adults

**DOI:** 10.3390/nu14163252

**Published:** 2022-08-09

**Authors:** Robert Barrett, Modar Youssef, Irfan Shah, Julia Ioana, Abdullah Al Lawati, Abdullah Bukhari, Suzanne Hegarty, Liam J. Cormican, Eoin Judge, Conor M. Burke, Catriona Cody, Joseph Feely, Katrina Hutchinson, William Tormey, Eoghan O’ Neill, Aoife O’ Shea, Meabh Connolly, Daniel M. A. McCartney, John L. Faul

**Affiliations:** 1 School of Biological and Health Sciences, Technological University Dublin, D08 NF82 Dublin, Ireland; 2 Department of Respiratory and Sleep Medicine, Connolly Hospital Dublin, D15 X40D Dublin, Ireland; 3 Department of Medicine, University College Dublin, D04 V1W8 Dublin, Ireland; 4 Department of Intensive Care Medicine, Connolly Hospital Dublin, D15 X40D Dublin, Ireland; 5 Department of Biochemistry, Connolly Hospital Dublin, D15 X40D Dublin, Ireland; 6 Eurofins Biomnis, Sandyford, Co. Dublin, D18 A4CO Dublin, Ireland; 7 Department of Microbiology, Connolly Hospital Dublin, D15 X40D Dublin, Ireland; 8 Department of Medicine, Royal College of Surgeons in Ireland, D02 YN 77 Dublin, Ireland

**Keywords:** COVID-19, vitamin D, hospitalization, mortality, Ireland

## Abstract

COVID-19 and a low vitamin D state share common risk factors, which might explain why vitamin D deficiency has been linked with higher COVID-19 mortality. Moreover, measures of serum vitamin D may become lower during systemic inflammatory responses, further confounding the association via reverse causality. In this prospective study (recruited over 12 months), we examined whether the association between a low vitamin D state and in-hospital mortality due to SARS-CoV-2 pneumonia in unvaccinated subjects is explained by (i) the presence of shared risk factors (e.g., obesity, advanced age) or (ii) a reduction in serum 25(OH)D due to COVID-19 (i.e., reverse causality). In this cohort of 232 (mean age = 56 years) patients (all had SARS-CoV-2 diagnosed via PCR AND required supplemental oxygen therapy), we failed to find an association between serum vitamin D and levels of CRP, or other inflammatory markers. However, the hazard ratio for mortality for subjects over 70 years of age (13.2) and for subjects with a serum 25(OH)D level less than 30 nmol·L^−1^ (4.6) remained significantly elevated even after adjustment for gender, obesity and the presence of diabetes mellitus. Subjects <70 years and >70 years had significantly higher mortality with a serum 25(OH)D less than 30 nmol·L^−1^ (11.8% and 55%), than with a serum 25(OH)D greater than 30 nmol·L^−1^ (2.2% and 25%). Unvaccinated Caucasian adults with a low vitamin D state have higher mortality due to SARS CoV-2 pneumonia, which is not explained by confounders and is not closely linked with elevated serum CRP.

## 1. Introduction

Retrospective studies [1,2] and studies that include only elderly subjects [3] or that include both inpatients and outpatients [4] or populations of both infected and uninfected subjects [5] have linked a low vitamin D state with both infection and mortality due to SARS-CoV-2 pneumonia [6]. Moreover, COVID-19 mortality appears to be higher in populations in whom vitamin D levels tend to be lower [7,8,9]. These observations might simply be attributable to the presence of confounders, since the risk factors for a low vitamin D state overlap with the main susceptibility factors for COVID-19 (e.g., advanced age, obesity and darker skin pigmentation) [10]. In addition, observed associations between a low vitamin D status and increased severity of COVID-19 disease might be affected by the vitamin D lowering effects of chronic corticosteroid therapy [11] or periods of pronounced systemic inflammation (i.e., reverse causality) [12]. Measures of serum 25(OH)D progressively fall in healthy human subjects injected with LPS (lipopolysaccharide), a reduction that appears to be related to the inflammatory response [12]. Therefore, the observation of low vitamin D levels in patients who die from COVID-19 may be explained by a bystander effect of systemic inflammation (reverse causality), or the presence of confounding factors, rather than a low vitamin D state having a causative role in COVID mortality [12]. Based on our own experience of poor clinical outcomes in patients who have low vitamin D status [13], we wanted to perform a prospective study of unvaccinated patients hospitalized with SARS-CoV-2 pneumonia. In particular, we wanted to study whether the association of low vitamin D status with mortality might be explained by patient selection bias (i.e., the presence of confounders) or by the presence of systemic inflammation (which might lower serum vitamin D measures). In order to eliminate the effect that the use of corticosteroids might have on our measures of both serum vitamin D and CRP, we did not recruit patients on chronic systemic corticosteroid therapy. We hypothesized that a low vitamin D state in hospitalized patients with SARS-CoV-2 pneumonia is associated with increased in-hospital mortality. 

## 2. Study Design and Methods

The study was conducted in accordance with the Declaration of Helsinki, and the protocol was approved by the Research Ethics Committee of Connolly Hospital Dublin. All subjects (except those on chronic systemic corticosteroid therapy) were eligible to enroll in a prospective observational study examining serum 25(OH)D and CRP levels and COVID disease severity. The cohort was defined as the patient population admitted to our hospital with COVID-19 pneumonia between March 2020 and April 2021. From a total of 972 hospitalized patients, 232 agreed to enroll (Table 1). To assess the prognostic value on mortality, we used the clinical scenario of a patient with COVID-19 with severe but not critical disease (i.e., patients with pneumonia, requiring oxygen support, but not invasive mechanical ventilation and/or hemodynamic support requirement). All had abnormal chest radiographs and a requirement for supplementary oxygen (O_2_). The primary outcome was mortality. We also measured length of admission, duration of oxygen therapy and admission to the intensive care unit. After signing an IRB approved consent form, background data including age, gender, body mass index (BMI) and comorbidities were recorded. The investigating team believed that patients who take chronic corticosteroid therapy might have lower measures of CRP, lower measures of 25(OH)D and increased mortality due to COVID pneumonia, and these patients were not recruited into the study.

Venous blood was collected into BD Vacutainer tubes^®^ containing EDTA, sodium citrate and no additive. Whole blood with EDTA was analysed for full blood count (FBC) using a Beckman Coulter UniCel^®^ DxH 800 automated analyser (DxH 800; Miami, FL, USA); the D-dimer levels were assessed on a Sysmex CS-2500 analyser with a Siemens kit (Siemens Healthcare Diagnostics, Marburg, Germany) on the day of collection. Total serum 25(OH)D levels were analysed on a Roche Cobas 6000′s module e602 (Roche Diagnostics GmbH, Mannheim, Germany) using an electrochemiluminescence immunoassay method with between-run and within-run CVs <8.9%. C-reactive protein (CRP), ferritin, troponin and total calcium and magnesium were measured, using commercially available diagnostic kits on the Roche Cobas 6000 automated analyser. The between-run and within-run CVs for these assays ranged between 1% and 8%. 

Biochemical (including serum 25(OH)D levels) and haematological laboratory parameters during the hospital stay were measured and the peak result was recorded. Serum 25(OH)D levels were measured and recorded within 24 h of admission. SARS-CoV-2 infection was confirmed in all patients using reverse transcription-polymerase chain reaction (RT-PCR) on samples which were obtained from nasopharyngeal swabs. 

We hypothesized that a serum 25(OH)D level less than 30 nmol·L^−1^ is associated with an increased risk of death within 30 days of admission. Our sample size was based on our estimated 20% mortality rate in hospitalized patients with SARS-CoV-2 pneumonia [13] and we anticipated a 95% confidence interval of 15% to 25% mortality. The required sample size was 246. Although the national prevalence of low vitamin D state with a serum 25(OH)D <30 nmol·L^−1^ is approximately 7% [14], we assumed that approximately 30% of subjects would have this low level, given the previously observed low levels of 25(OH)D in our patients who required mechanical ventilatory support for COVID-19 pneumonia (12 of 33 patients < 30 nmol·L^−1^) [13].

## 3. Statistical Analysis

All statistical analyses were performed using SPSS statistical software (SPSS statistics for MacOS, IBM Corp, Version 27.0, 64-bit edition, Armonk, NY, USA). Categorical variables were expressed as absolute numbers (*n*) and relative frequencies (%). Continuous variable data were tested for normality using Shapiro—Wilk’s test, a visual inspection of their histograms, and via assessment of their skewness and kurtosis. For all analyses, a *p* value of <0.05 was considered statistically significant.

Descriptive statistics were used to define patients’ demographic and comorbidity characteristics. Vitamin D status was divided into three categories: D30 as a serum 25(OH)D concentration <30 nmol·L^−1^, D40 was defined as a serum 25(OH)D concentration of 30–49.9 nmol·L^−1^, while D50 was defined as a serum 25(OH)D concentration ≥50 nmol·L^−1^. Parametric and non-parametric analyses were used to compare various laboratory biomarkers between COVID-19 patients in the three vitamin D categories. For normally distributed continuous variables, mean levels and standard deviation (SD) were compared between the three groups using analysis of variance (ANOVA) tests; for non-normally distributed continuous variables, median levels and interquartile ranges (IQR) were compared between the three groups using Kruskal—Wallis tests.

Pearson’s Chi-square test was used to assess the association between the three vitamin D status categories and outcome measures such as extended duration of O2 requirement (i.e., >24 h), ICU admission and mortality. Binary logistic regression analysis was used to determine if age, gender, presence of diabetes mellitus, obesity (BMI ≥ 30 kg.m−2) or renal disease predicted the likelihood of having a 25(OH)D level < 30 nmol·L−1. 

Multivariate logistic regression analyses were finally used to assess whether vitamin D deficiency (25(OH)D < 30 nmol·L^−1^) predicted clinical outcomes such as O_2_ requirement >24 h, ICU admission and mortality after controlling for confounders. Study participants were dichotomised into D30 (<30 nmol·L^−1^) and other (≥30 nmol·L^−1^) groups for these analyses. 

Univariate analyses were used to determine whether serum measures of CRP or clinical outcome (e.g., O_2_ requirement >24 h, ICU admission, mortality) were associated with measures of 25(OH)D. Multivariate logistic regression analyses were used to determine whether any observed associations between background status (age, BMI, diabetes mellitus etc.) and 25(OH)D and between 25(OH)D and clinical outcomes persisted after adjusting for confounders. We performed additional analyses, including Yates’ continuity correction, to detect differences in mortality rates with a 2 × 2 crosstabulation of mortality in the over 70 years group and the under 70 years age groups vs. (<30 nmol/L) and (>30 nmol/L) serum 25(OH)D measurements. A Mann—Whitney U test was employed to detect significant differences in serum 25(OH)D levels between survivors and non-survivors in older and younger age groups. 

## 4. Results

The socio-demographic, anthropometric and clinical characteristics of the patient cohort are summarised in Table 1. The median age of participants was 56 years (range 17–99), and approximately 60% of the patients were male. In relation to vitamin D status, 38% (*n* = 88) of participants were D30 (25(OH)D <30 nmol·L^−1^), 26% (*n* = 60) were D40 (25(OH)D of 30–49 nmol·L^−1^), while 36 % (*n* = 84) were D50 (25 (OH) ≥ 50 nmol·L^−1^).

The biometric associations between serum 25 (OH) and other biochemical and hematological indices are described in Table 2. Higher median CRP levels were observed in the D30 (114 mg·L ^−1^) and D40 (158 mg·L^−1^) groups than in the D50 group (91 mg·L ^−1^), although this trend did not reach statistical significance (*p* = 0.39) (Table 2). Median D-dimer levels were also higher amongst the D30 group (895) and D40 group (902) than the D50 group (768), although again, this trend did not reach statistical significance (*p* = 0.45). There was a statistically significant difference in lymphocyte count across the three vitamin D groups (*p* = 0.036). Lymphocyte levels were higher in the D50 group (1 × 10^9^) and the D30 group (1 × 10^9^) than in the D40 group (0.68 × 10^9^) (*p* = 0.016 and *p* = 0.026, respectively) (Table 2).

Regarding clinical outcomes, 56% of those in the D50 sufficient group required supplemental oxygen therapy for more than 24 h, compared with 70% and 75% in the D40 and D30 groups, respectively, though this trend did not reach statistical significance (*p* = 0.249) (Table 3). Both the D40 group (28%) and the D30 group (22%) had a significantly higher likelihood of ICU admission than the D50 group (9.5%) (*p* = 0.013). Additionally, significantly lower median 25(OH)D concentrations were seen in patients who required ICU admission (33 nmol·L^−1^) compared to those who did not require ICU admission (42 nmol·L^−1^) (*p =* 0.031). Subjects in the D30 group experienced higher mortality (22%) than patients in either the D40 group (6.5%) or the D50 group (13%) on univariate analysis (*p* = 0.037) (Table 3).

Binary logistic regression analysis was performed to assess the association between a set of putative predictor variables and the likelihood of a 25(OH)D ≤ 30 nmol·L^−1^ (Table 4). The model comprised five independent variables (gender, age, diabetes mellitus status, obesity (BMI ≥30 kg.m^−2^) and renal disease). As shown in Table 4, those aged ≥70 years were significantly less likely to have a 25(OH)D ≤ 30 nmol·L^−1^ (OR: 0.520 (95% CI: 0.285–0.951) (*p* = 0.034)), but none of the other parameters was independently associated with a 25(OH)D ≤ 30 nmol·L^−1^.

Multivariate logistic regression was performed to assess the association between vitamin D status and clinical outcomes after adjustment for the potential confounders included in Table 5. After adjusting for age, gender, presence of diabetes and obesity, a 25(OH)D < 30 nmol·L^−1^ was strongly predictive of mortality (OR: 4.63 (95% CI: 1.53–13.97) (*p* = 0.006)). Age ≥70 was also a strong predictor of mortality (OR: 13.32 (95% CI: 4.24–41.81) (*p* < 0.001)). Diabetes mellitus, male gender and obesity were not independently associated with mortality.

Male gender (OR: 2.90 (95% CI: 1.29–6.51) (*p* = 0.01)) and diabetes mellitus (OR: 2.82 (95% CI: 1.21–6.53) (*p* = 0.016)) were independent predictors of ICU admission however. A negative association between age ≥70 years (OR: 0.29 (95% CI: 0.11–0.73) (*p* = 0.009)) and ICU admission was also observed. A measure of 25(OH)D <30 nmol·L^−1^ was not a significant predictor of ICU admission (OR: 1.18 (95% CI: 0.58–2.39) (*p* = 0.632)). After adjusting for male gender, 25(OH)D <30 nmol·L^−1^ was not a significant predictor of supplemental O_2_ >24 h (OR: 2.11 (95% CI: 0.85–5.39) (*p* = 0.116)). Male gender itself however was a significant predictor of requirement for supplemental O_2_ >24 h (OR: 3.22 (95% CI: 1.26–8.22) (*p* = 0.014)).

## 5. Discussion

In this prospective, observational study of unvaccinated Caucasian patients hospitalized for SARS-CoV-2 pneumonia, a serum 25(OH)D lower than 30 nmol·L^−1^ was associated with a greater than four-fold increased mortality risk. The observed association is not easily explained by differences in race (all subjects were Caucasian) and the effect persisted after allowing for gender, BMI, and the presence of diabetes mellitus. While uncertainty exists about what measures accurately reflect vitamin D status, here we employed 25(OH)D because it is in widespread clinical use and is used in many epidemiologic studies, although measures of 25(OH)D may not accurately reflect vitamin D status of patients who receive large bolus injections of vitamin D2 [15]. Nevertheless, our data support the idea that vitamin D plays a causative role in the progression of COVID [14,16]. 

Here, we confirm that advancing age (greater than 70 years) was a significant independent risk for mortality with a hazard ratio of greater than 13. Indeed, the mean age of our population was 56 years, which may explain why the mortality rate in our study (15%) is somewhat lower than that reported in other prospective studies. For example, in one ICU study with a mean age of 65 years, the 28 day mortality was 33% for subjects with 25(OH)D measures less than 38 nmol·L^−1^ [17]. The mortality rate in the control group (mean age = 66 years) of the RECOVERY trial (i.e., those who required oxygen therapy without respiratory support at the time of randomization) was also higher at 26%, supporting the idea that advancing age increases mortality from COVID pneumonia [18].

Our decision to not recruit patients prescribed systemic corticosteroid therapy is supported by a recent large study of 7845 hospitalized patients, in whom corticosteroid therapy alone (within 2 weeks of a positive SARS-CoV-2 PCR test) was associated with significantly higher mortality (17%), compared to the mortality of subjects who took neither vitamin D supplements nor corticosteroids (12.5%) [19]. Interestingly, hospitalized subjects in that study who took vitamin D alone (without corticosteroids) also demonstrated a significantly lower mortality (6%) [19]. Our finding of a negative association of age and ICU admission but a positive association of age with mortality might be explained by a rapid deterioration to death prior to ICU admission in some frail elderly patients, although we did not quantify frailty. 

The strength of this study is that we have prospectively recruited unvaccinated patients with their first episode of COVID pneumonia at a single centre and we did not recruit subjects who took systemic corticosteroid therapy, because of the apparent reduction in measures of serum 25(OH)D and CRP seen in such subjects [11,20]. We avoided the use of the term “vitamin D deficient” because that term is most commonly used in relation to bone health [21]. Because very few (only four) of our subjects had a 25(OH)D level greater than 100 nmol·L^−1^ and none of those died, we cannot make a meaningful comment about the level of 25(OH)D, if any, where immunocompetent adults are protected from severe COVID. Indeed, many of our subjects with a level greater than 50 nmol·L^−1^ suffered pneumonia, respiratory failure and death due to COVID, indicating that some subjects with a serum 25(OH)D significantly greater than 30 nmol·L^−1^ do suffer severe COVID.

While oral vitamin D supplementation data for the current cohort are unavailable, data from the Irish National Adult Nutrition Survey (NANS) in 2009 show a steady age-related increase in the use of vitamin D supplements from the 18–24 year age group (10% of men and 12% of women) to the 50–64 year age group (18% of men and 27% of women) [22]. In older Irish adults, vitamin D-containing supplement use has been estimated at 33.5% in one cohort of community-dwelling Irish adults aged >60 years [23], with the National Adult Nutrition Survey estimating the prevalence of vitamin D supplementation at 27.6% in adults aged >65 years vs. 16.0% of adults aged 18–64 years [24]. In older Irish adults, vitamin D supplementation has been identified as a potent predictor of vitamin D status, with supplement use associated with an incremental rise in serum 25(OH)D of between 24 and 27 nmol^−1^ in these older adults [23,25]. 

Recent data suggest that supplementation rates (and by extrapolation vitamin D levels) pre-admission to hospital are significantly higher in subjects with less severe disease [26]. Since we used 25(OH)D levels on recruitment to the study (the day of admission to hospital), we have no information on 25(OH)D levels in the months preceding infection. In Ireland, the time of year (season) significantly influences serum 25(OH)D levels, and lower or higher levels might have been measured in the weeks prior to infection [24]. Our recruitment period spanned 12 months, mitigating the effect of season on the outcomes. 

Our data are consistent with the results of a number of prospective observational studies of hospitalized COVID-19 patients that also demonstrate lower vitamin D levels are significantly associated with 28 day mortality [27,28], in particular, two recent meta-analyses: one review of 14 studies comprising 999,179 participants that found low serum 25(OH)D is associated with higher COVID-19 mortality, with an odds ratio of 3.08 [9], and a second systematic review and meta-analysis of 43 studies that also describes low vitamin D state associated with higher COVID-19 mortality [28]. Our D30 patients were 4.63 times more likely to die than their vitamin D replete peers (*p* = 0.006), even after adjustment for age, gender, presence of diabetes mellitus and BMI. In a German study of hospitalised patients, even after adjustment for age, gender and underlying disease, the risk of mechanical ventilation was 6.1 times higher and risk of death was 14.7 times higher in those with 25(OH)D < 30 nmol·L^−1^ than in those with 25(OH)D > 30 nmol·L^−1^ (*p* < 0.001 for both) [4]. In India, mean serum 25(OH)D was 70 nmol·L^−1^ in asymptomatic SARS-CoV-2 positive patients versus 36 nmol·L^−1^ in severely-ill COVID-19 patients requiring ICU admission (*p* = 0.0001) [29]. Vitamin D deficiency (defined in that study as 25(OH)D < 50 nmol·L^−1^) was present in 97% of patients requiring ICU admission versus 33% of asymptomatic patients, with a case fatality rate of 21% in those with 25(OH)D < 50 nmol·L^−1^ vs. 3.1% in those with 25(OH)D > 50 nmol·L^−1^ [29]. In Boston and New York, requirements for invasive mechanical ventilation and in-hospital mortality similarly declined with increasing vitamin D status, even after adjustment for confounders [8]. In contrast, a number of genetically predicted and baseline vitamin D studies fail to show an association between lower 25(OH)D levels and COVID-19 mortality, suggesting environmental factors might play a role in modifying 25(OH)D levels prior to developing COVID [30,31]. It is unsurprising that we found only a non-significant trend towards higher CRP and D-dimer levels in subjects with lower 25(OH)D levels, and we also failed to find other associations between serum 25(OH)D and the markers of inflammation and thrombophilia which typically characterize severe COVID-19, because all patients recruited to the study had severe disease at baseline (SARS-CoV-2 pneumonia requiring supplemental oxygen). 

**Figure 1:**
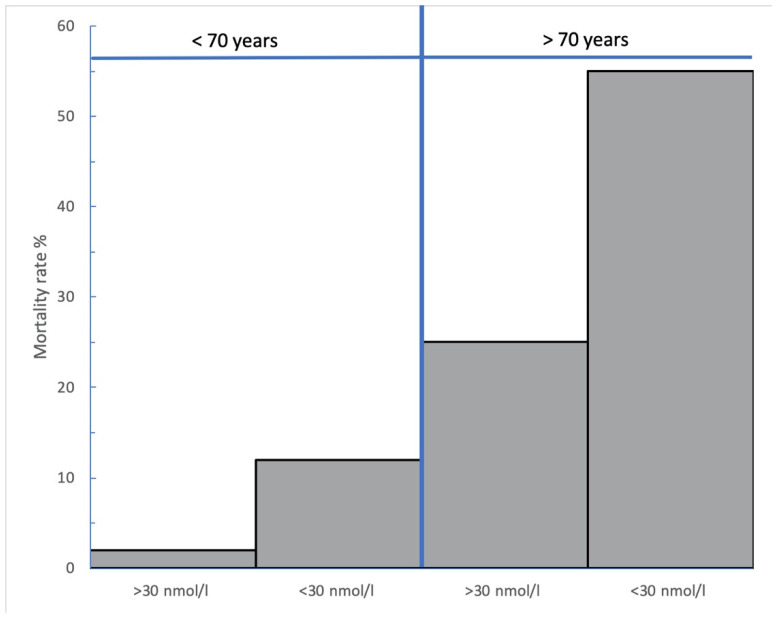
Mortality rates according to age and vitamin D status. The x axis denotes serum vitamin D level (25(OH)D level in nmol·L^−1^) for subjects younger than 70 years and older than 70 years. The y axis represents % mortality rate.

The mortality rate (10/160, 6.3%) for subjects younger than 70 years was lower than for subjects over 70 years of age (24/72 = 33.3%). Although this study was not designed to compare younger and older subjects, we made some interesting observations: First, in the subjects aged 70 years and over, overall mortality was 33% (24/72), but was significantly higher for subjects with a serum 25(OH)D < 30 nmol·L^−1^ (11/20, 55%) than in those with serum 25(OH)D ≥ 30 nmol·L^−1^ (13/52, 25%) (*p* = 0.032) (Figure 1). Overall, subjects who were aged over 70 years and who also had a serum 25(OH)D level less than 30 nmol·L^−1^ had substantially higher mortality (11/20, 55%) compared to the remainder of the study population (23/212, 10.8%) (*p* < 0.001) (Figure 1). Conversely, mortality rates for our subjects who were both under 70 years of age AND had 25(OH)D levels >30 nmol·L^−1^ was only 2.2% (2/92 patients)—significantly lower than the 22.9% mortality rate (32/140 patients) observed amongst the remainder of the study population (*p* < 0.001). Thus, in this study population, patients aged >70 years with 25(OH)D levels <30 nmol·L^−1^ represented just 8.6% of the overall cohort, but accounted for one third of the deaths. This finding is mirrored in the analysis of younger subjects: in our study population, younger than 70 years with a serum 25(OH)D <30 nmol·L^−1^ mortality was significantly higher (8/68 = 11.8%) compared to the population of patients aged less than 70 years with 25(OH)D >30 nmol·L^−1^ (2/92 = 2.2%) (*p* = 0.032) (Figure 1). Second, and consistent with prior population studies of Irish adults [23,25], serum 25(OH)D levels were higher in our subjects aged >70 years (55.9 SD ± 32.7 nmol·L^−1^) compared to those under 70 years (42.8 SD ± 28.1 nmol·L^−1^) (*p* = 0.002). We did not specifically study supplementation rates, but it is likely that higher oral supplementation rates and greater time spent outdoors in summertime might account for some of the difference. Interestingly, the average measures of serum 25(OH)D in survivors over the age of 70 years (56.9 ± 29.3 nmol·L^−1^) did not differ significantly from those over 70 years who died (54.0 ± 39.1 nmol·L^−1^) (*p* = 0.729). In contrast, the mean serum 25(OH)D was significantly lower in subjects younger than 70 years who died (mean 24.5 ± 12.4 nmol·L^−1^) compared to subjects younger than 70 years who survived (mean 44.1 ± 28.4 nmol·L^−1^) (*p* = 0.032).

A low serum vitamin D state has been proposed in some studies to arise as a consequence of systemic inflammation (i.e., reverse causality). For example, Smolders et al. demonstrated small but significant drops in serum 25(OH)D (~6 nmol·L^−1^) in healthy subjects who were injected with LPS (a surrogate for gram-negative septic shock syndrome) [12]. Although the reduction is modest, one might expect much larger drops in serum vitamin D levels during the course of a long illness. Thus, a low vitamin D level might be associated with greater disease severity only because vitamin D is consumed during the inflammatory processes of severe COVID-19. The current data suggest that the observed associations between low serum 25(OH)D and severe clinical outcome are not attributable to any depletive effect of the disease process itself, since measurements of 25(OH)D levels were not significantly associated with measures of CRP or D-dimers. Elevations in CRP and D-dimer have previously been associated with increased disease severity and mortality in broader COVID-19 patient populations (i.e., those that include subjects with mild disease), with some studies revealing an association between low vitamin D status and these pro-inflammatory, pro-thrombotic signatures [32,33,34]. Consistent with this literature, we found that in this subset of patients hospitalized with pneumonia, CRP and D-dimer levels were high in all subjects (albeit the measures were somewhat higher in the D30 group and the D40 group than in the D50 group). Raised D-dimer levels have been associated with the dysregulated coagulation seen in COVID-19 and are thought to predict increased disease severity. In a large (non-COVID) population cohort study, Hypponen et al. found that serum 25(OH)D concentrations were inversely associated with D-dimer [35]. A recent meta-analysis described thrombocytopenia as being associated with a five-fold increased risk of severe COVID-19 and mortality [36]; however, we found no difference in mean platelet count according to vitamin D status.

This prospective study demonstrates that a low vitamin D state is associated with increased mortality rates in patients with COVID pneumonia—and that this effect is not confined to older age groups. There appears to be an incremental rise in mortality rates in both younger and older age groups according to vitamin D status and age. In this regard, a recent meta-analysis incorporating data from eight studies in which 25(OH)D was measured prior to infection or on the first day of hospitalisation has shown a significant inverse correlation between these pre- and peri-infection 25(OH)D levels and mortality, proposing a threshold for minimal mortality risk at ~125 nmol·L^−1^ [37]. This study included very few (*n* = 4) subjects with 25(OH)D levels greater than 100 nmol·L^−1^, and while none died, we cannot make any comment about what level, if any, protects against COVID mortality. Nevertheless, our data add to a growing body of literature which now meets many of the Bradford Hill criteria supporting a causal link between low vitamin D status and COVID-19 disease severity and death [38].

The current data suggest that unvaccinated subjects, who become hospitalized with COVID pneumonia, have increased mortality with increasing age and with lower serum 25(OH)D levels. Vitamin D has a multitude of effects on the human immune system. Metabolites of vitamin D (e.g., 1,25 (OH)_2_ D_3_) impact cellular function via both nuclear receptor-dependent and nuclear receptor-independent mechanisms [34]. The nuclear receptor dependent pathways involve 1,25 (OH)_2_ D_3_-bound vitamin D receptors (VDR) forming a heterodimer complex with retinoid X receptors (RXRA, RXRB, RXRG) and subsequently binding to vitamin D response elements regulating gene transcription [39]. In contrast, the rapid-onset nuclear receptor-independent effects of 1,25 (OH)_2_ D_3_ are mediated by a membrane-associated rapid response steroid-binding protein, identified as PDIA3, with impacts on cell survival and immune responses [40,41]. If vitamin D has immunological effects that are relevant to the progression of SARS-Cov-2 [41], then the observed association between a low vitamin D state and increased COVID mortality should be more apparent in unvaccinated than vaccinated subjects (because vaccination amplifies adaptive responses to viral infection). In such a scenario, vitamin D supplementation may play a vital role in protecting both unvaccinated patients and patients in whom the effect of vaccination wanes [42].

## Figures and Tables

**Table 1:** Socio-demographic, anthropometric and clinical status of COVID-19 patients (*n* = 232). Median age 56 years (range 17–99).

Male	60 (%)	
**BMI (kg/m^2^) (%)** <18.5 kg/m^2^ 18.5–24.9 kg/m^2^ **25–29.9 kg/m^2^** **≥30 kg/m^2^**	(%) 4 36 22 38	
Current **Smoker**	2%	
**Comorbidity** Diabetes Mellitus Hypertension **Hyperlipidaemia** **Renal Disease** **Respiratory Disease** **Malignancy** **Other Comorbidity**	*n* (%) 16 35 15 5 23 10 49	
**Serum 25(OH)D (nmol·L^−1^) (%)** Deficient (<30 **nmol·L^−1^**) Insufficient (30–49.9 **nmol·L^−1^**) Sufficient (>50 **nmol·L^−1^**)		(%) 38 26 36
**Mortality (%)** Survivors Non-survivors		*n* (%) 85 15
**ICU Admission (%)** Admitted to ICU > 24 h		*n* (%) 19
**Extended O_2_ Requirement > 24 h (%)** Yes		*n* (%) 69

Body mass index (BMI), intensive care unit (ICU), oxygen (O_2_).

**Table 2:** Laboratory parameters of COVID-19 patients in relation to vitamin D status. Peak measures during hospital admission are reported. Serum 25(OH)D was measured within 24 h of admission.

Parameter *Biochemistry*	25(OH)D Concentration < 30 nmol·L^−1^ (Deficient)	25(OH)D Concentration 30–49.9 nmol·L^−1^ (Insufficient)	25(OH)D Concentration > 50 nmol·L^−1^ (Sufficient)	*p* Value
CRP (mg/L) (median (IQR))	114 (182)	158 (153)	91 (148)	0.390
WBC (C/mL) (mean (SD))	7.43 × 10^9^ (3.89 × 10^9^)	6.42 × 10^9^ (2.84 × 10^9^)	7.74 × 10^9^ (2.74 × 10^9^)	0.409
Lymphocytes (C/mL) (median (IQR))	1.00 × 10^9^ (0.63 × 10^9^)	0.68 × 10^9^ (0.47 × 10^9^)	1.00 × 10^9^ (0.56 × 10^9^)	0.036
Eosinophils (C/mL) (median (IQR))	0.00 × 10^9^ (0.30 × 10^9^)	0.00 × 10^9^ (0.01 × 10^9^)	0.00 × 10^9^ (0.05 × 10^9^)	0.554
Neutrophils (C/mL) (mean (SD))	5.62 × 10^9^ (3.5 × 10^9^)	5.12 × 10^9^ (2.76 × 10^9^)	5.83 × 10^9^ (2.49 × 10^9^)	0.739
Ferritin (ng/mL) (median (IQR))	894 (1250)	814 (1944)	1068 (1663)	0.929
D-Dimer (ng/mL) (median (IQR))	895 (3039)	902 (806)	768 (1513)	0.453
Platelets (10^9/^L) (mean SD))	216 (88)	219 (119)	222 (70)	0.965
Troponin (ng/mL) (median (IQR))	6.5 (13.2)	6.7 (11.2)	7.0 (9.7)	0.923
ACE-2 (ng/mL) (median (IQR))	35 (25)	32 (12)	35 (48)	0.788
Calcium (mmol/L) (median (IQR))	2.19 (0.18)	2.20 (0.19)	2.20 (0.2)	0.180
Magnesium (mmol/L) (median (IQR))	0.86 (0.16)	0.82 (0.11)	0.83 (0.13)	0.119

Normally distributed continuous variables are expressed as mean and standard deviation (SD), while non-normally distributed continuous variables are expressed as median and interquartile range (IQR). Interquartile range (IQR), standard deviation (SD), C-reactive protein (CRP), white blood cell (WBC), angiotensin converting enzyme 2 (ACE-2).

**Table 3:** Clinical outcomes of patients according to vitamin D status (*n* = 232).

Parameter	25(OH)D Concentration <30 nmol/L (*n* = 88)	25(OH)D Concentration 30–50 nmol/L (*n* = 60)	25(OH)D Concentration >50 nmol/L (*n* = 84)	*p* Value
	*n* (%)	*n* (%)	*n* (%)	
O_2_ Requirement >24 h	36 (75)	14 (70)	14 (56.0)	0.249
ICU Admission >24 h	19 (21.6)	17 (28.3)	8 (9.5)	0.013
Mortality Non-survivors	19 (22)	4 (7)	11 (13)	0.037

Categorical variables are expressed as their total number *n* and percentages (%), while non-normally distributed continuous variables are expressed as median (Md) and interquartile range (IQR). Interquartile range (IQR), oxygen (O_2_), intensive care unit (ICU).

**Table 4:** Binary logistic regression analysis of putative factors associated with serum 25(OH)D concentration <30 nmol/L.

Category	Beta Coefficient	Standard Error (SE)	OR (95% CI)	*p* Value
Gender	0.069	0.277	1.071 (0.622–1.844)	0.804
Age	−0.653	0.308	0.520 (0.285–0.951)	0.034
Diabetes Mellitus	0.053	0.373	1.055 (0.508–2.189)	0.886
Renal Disease	0.338	0.937	1.402 (0.223–8.801)	0.718
BMI (≥30 kg/m2)	0.382	0.358	1.465 (0.726–2.959)	0.286

Reference categories: female gender, age <70 years, absence of diabetes mellitus, absence of renal-disease, BMI < 30 kg/m2, 25(OH)D concentration ≥30 nmol/L. Odds ratio (OR), confidence interval (CI), body mass index (BMI).

**Table 5:** Multivariable logistic regression analyses predicting mortality, ICU admission and O_2_ requirement with 25(OH)D status < 30 nmol/L while controlling for confounding factors.

Outcome Measure	Covariate	OR (95% CI)	*p* Value
Mortality	25(OH)D Concentration <30 nmol/L	4.63 (1.53–13.97)	0.006
	Age ≥70 years Diabetes Mellitus Male Gender Obesity	13.32 (4.24–41.81) 1.59 (0.43–5.86) 0.90 (0.33–2.41) 2.03 (0.744–5.57)	<0.001 0.484 0.836 0.166
ICU Admission	25(OH)D Concentration < 30 nmol/L	1.18 (0.58–2.39)	0.632
	Age ≥70 years	0.29 (0.11–0.73)	0.009
	Male Gender	2.90 (1.29–6.51)	0.010
	Diabetes Mellitus	2.82 (1.21–6.53)	0.016
Extended O_2_ Requirement >24 h	25(OH)D Concentration < 30 nmol/L	2.11 (0.83–5.39)	0.116
	Male Gender	3.22 (1.26–8.22)	0.014

Reference categories: 25(OH)D concentration ≥30 nmol/L, age <70 years, absence of diabetes mellitus, female gender. Odds ratio (OR), confidence interval (CI), intensive care unit (ICU), oxygen (O_2_).

## Abbreviations

25(OH)D	25-Hydroxyvitamin Vitamin D
ARDS	Acute Respiratory Distress Syndrome
BMI	Body Mass Index
COVID-19	Coronavirus Disease 2019
CRP	C-Reactive Protein
HR	Hazard Ratio
ICU	Intensive Care Unit
n	Total number of cases
O_2_	Oxygen
OR	Odds Ratio
PaO_2_ RXR	Partial Pressure of Oxygen Retinoid X Receptor
SARS-CoV-2	Severe Acute Respiratory Syndrome Coronavirus-2
WBC	White Blood Cell
μg	Microgram
